# Martini Coarse-Grained Model of Hyaluronic Acid for the Structural Change of Its Gel in the Presence of Monovalent and Divalent Salts

**DOI:** 10.3390/ijms21134602

**Published:** 2020-06-29

**Authors:** Raj Kumar, Young Kyu Lee, Yong Seok Jho

**Affiliations:** 1 Department of Physics and Research Institute of Natural Science, Gyeongsang National University (GNU), 501 Jinju-daero, Jinju 52828, Korea; raj.kumar@juit.ac.in (R.K.); monsterplank@gmail.com (Y.K.L.); 2 Department of Biotechnology and Bioinformatics, Jaypee University of Information Technology (JUIT), Waknaghat, Solan 173234, India

**Keywords:** hyaluronic acid, gel formation, coarse-grained simulation, Martini

## Abstract

Hyaluronic acid (HA) has a wide range of biomedical applications including the formation of hydrogels, microspheres, sponges, and films. The modeling of HA to understand its behavior and interaction with other biomolecules at the atomic level is of considerable interest. The atomistic representation of long HA polymers for the study of the macroscopic structural formation and its interactions with other polyelectrolytes is computationally demanding. To overcome this limitation, we developed a coarse grained (CG) model for HA adapting the Martini scheme. A very good agreement was observed between the CG model and all-atom simulations for both local (bonded interactions) and global properties (end-to-end distance, a radius of gyration, RMSD). Our CG model successfully demonstrated the formation of HA gel and its structural changes at high salt concentrations. We found that the main role of CaCl_2_ is screening the electrostatic repulsion between chains. HA gel did not collapse even at high CaCl_2_ concentrations, and the osmotic pressure decreased, which agrees well with the experimental results. This is a distinct property of HA from other proteins or polynucleic acids which ensures the validity of our CG model. Our HA CG model is compatible with other CG biomolecular models developed under the Martini scheme, which allows for large-scale simulations of various HA-based complex systems.

## 1. Introduction

Hyaluronic acid (HA), also called hyaluronan, is a linear anionic polysaccharide comprised of repeating disaccharide units of D-glucuronic acid (GlcA) and N-acetyl-D-glucosamine (GlcNAc) linked by alternating β(1–3) and β(1–4) glucosidic bonds [1]. HA is widely distributed and found in several locations in the body such as skin, synovial fluid, eyes, and lungs. HA is a component of the extracellular matrix (ECM), and its primary functions are to trap water inside tissue cells, maintain the moisture of eyes, and keep the joints well lubricated. HA can be chemically modified through conjugation and crosslinking polymerization processes, and its properties have been customized for several biomedical applications [2]. An interesting property of HA is that it keeps a liquid form in the physiological NaCl conditions, even under high CaCl_2_ concentrations, which is unusual for other biological polyelectrolytes. Due to this property and the high biocompatibility of HA, it has been widely investigated for the development of hydrogel scaffolds for tissue engineering [3]. The use of HA-based dermal fillers reduces the depth of skin folds and is used for facial rejuvenation in the area of cosmetic surgery. Different polyelectrolyte polymers have been studied in complex coacervate formation with HA. HA complex coacervation has also been investigated with different proteins such as silk fibroin and lysozyme. HA coacervate has been used to isolate the target protein bovine serum albumin from a mixture of β-lactoglobulin based on its electrostatic charge properties [4]. HA complexation stabilized the nanoparticles used in various gene silencing and gene therapy strategies. Several other formulations of covalently bound or cross-linked HA molecules forming hydrogel networks have been used for controlled-release drug delivery applications. HA polymers have been investigated in several other biomedical applications including tissue engineering, dermal filler for skin rejuvenation, visco-supplementation for arthritis, wound healing, ocular treatment, dermatology, plastic surgery, drug delivery, the delivery of therapeutic and biological competent cells, vaccine delivery, and gene therapy. Polyelectrolyte gels are considered as a model to study swelling behavior in several theoretical and applied research studies including muscle contraction, protein transitions, and nerve excitation [5,6,7]. Chemically crosslinked polyelectrolyte gels have greater swelling capacities than non-ionic polymer gels, which are advantageous in biomedical applications. Polyelectrolyte gel swelling is the subject of numerous studies in polymer physics which demonstrate that minute changes in external conditions such as temperature, external electric field, solvent composition, and ionic strength can induce drastic changes in gel swelling properties [8]. Exposure to multivalent counterions causes the precipitation of polyelectrolytes, which poses a huge problem in bioengineering [9]. However, HA does not precipitate at high multivalent counterion concentrations, which distinguishes this molecule from other biopolymers such as DNA [10]. This study correlates the behavior of HA gel formation in varying concentrations of the monovalent salt NaCl and the divalent salt CaCl_2_ from simulations to actual laboratory experiments.

A complex coacervation process is the binding of two oppositely charged molecules facilitated mainly by electrostatic interaction, ultimately leading to a liquid–liquid phase separation and coacervate formation [11]. Additionally, hydrophobic–hydrophobic interactions, van der Waals intermolecular force, and intermolecular hydrogen bonding also play important roles in the mechanism of gel formation such as complex coacervation. Several molecular simulation approaches, such as Monte Carlo, Langevin dynamics, and molecular dynamics simulations, can be used to understand this polyelectrolyte complexation [12,13]. A computational demonstration of a complex process of coacervate gel formation may require simulation time scales that are too large to be studied in atomistic detail. However, lowering the level of polymer representation from all-atom to coarse-grained (CG) opens up new possibilities for studying polymer complexation and its phase separation behavior [14]. CG models based on the Martini scheme have been used for commonly used synthetic polymers such as polycarbonates, polystyrene, polyamide, polyethylene, and polypropylene [15,16,17,18]. Further, coarse-grained studies of the self-assembly mechanism of Pluronic or poloxamer triblock copolymers reproducing experimental micelle sizes and shapes have been reported [19,20]. Martini-based models have also been applied to understand the cross interactions of protein–polymer complex formations [21]. Several recent reports justify the application of Martini CG models to a variety of molecules, including calcein fluorescent dye [22], polyethylenimine [23], clay–polymer nanocomposites [24], poly(3,4-ethylenedioxythiophene) (PEDOT) [25], etc. Moreover, Martini-based CG models have been successfully applied to study supramolecular polymer assemblies such as benzene-1,3,5-tricarboxamide (BTA) [26,27] and peptide amphiphiles [28,29]. Additionally, Martini parameters have been developed for biopolymers and a wide variety of biomolecules such as membrane lipids, glycolipids, carbohydrates, proteins, DNA, and RNA [30]. Continuous efforts for developing transferable Martini models have been reported for several molecules, including polyethylene oxide (PEO) [31], polyethers [32], perfluorosulfonic acid polymer membranes [33], and methacrylate-based copolymers [34]. Martini models have also been successful in studies capturing the phase behavior and phase transitions of several polymer systems [35,36,37,38,39,40,41]. The above examples justify the wide acceptability of Martini CG models for various compounds and polymer systems. In this study, we developed and validated a CG model for HA based on the Martini scheme for modeling coarse-grained simulations, which will make it possible to perform greatly accelerated simulations for understanding events in HA polyelectrolyte complexation. We applied our model to studies on the structural change of HA gel in the presence of monovalent and divalent salts, the microscopic mechanism of which is not yet clearly understood. Our computational studies rendered the microscopic mechanism of the previous experiments. The HA CG model presented here will make it possible to perform greatly accelerated simulations for understanding events in HA polyelectrolyte complexation.

## 2. Results and Discussion

### 2.1. Measures of CG Simulations

**Figure 1:**
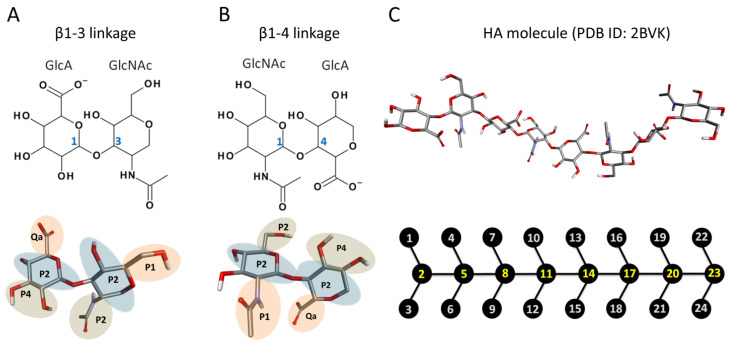
Chemical structures (top) and atomistic visualization (bottom) of HA1-3 and HA1-4 disaccharides (**A**,**B**). (**C**) Atomistic representation (top) of octasaccharide hyaluronic acid (HA) and its coarse-grained (CG) representation (bottom).

Here, we develop the CG model of hyaluronic acid (HA) by the basic principles described in the Methods section. HA is a linear polymer made up of repeating disaccharide units of GlcA and GlcNAc connected by β1-3 (HA1-3) and β1-4 (HA1-4) linkages (Figure 1). Therefore, we modeled two different bonding monosaccharide units separately and incorporated the obtained parameters to model a larger molecule of HA containing eight monosaccharide units [42].

#### 2.1.1. Matching AA and CG Bonded Distributions

Determining the parameters for bonded terms is an iterative process where initial values are first guessed, followed by tuning these values by trial and error. First, we obtained trajectories of all-atom (AA) simulations for all HA structures. All the AA trajectories were then converted to CG trajectories based on the CG mapping scheme described above. The probability distributions for the bonded terms could be calculated from these obtained CG trajectories which will be referred to as the AA distributions. The gmx distance program was used to calculate the bond distributions between averaged CG points obtained by averaging from AA simulations. The angles and dihedral distributions were calculated by using the gmx angle program. Secondly, a random guess for bonded terms was made to obtain initial parameters for running the CG simulations. The new probability distributions for bond lengths, angles, and dihedrals were calculated from the obtained CG trajectories, which are referred to as the CG distributions. The CG distributions were compared with the corresponding averaged AA distributions in order to obtain a good match. In case of a mismatch, CG parameters were continuously updated to reduce the distance between the two sets of distributions until a good match was obtained.

#### 2.1.2. Global Properties

Global properties of HA polymer such as the end-to-end distance (R_e_) and radius of gyration (R_g_) were calculated for both AA and CG distributions (Equation (1)). The R_e_ and R_g_ were calculated by using the gmx polystat program which plots the static properties of polymers as a function of time and prints the average value.

(1) $$R_{g}=\left(\frac{\sum_{i}||r_{i}|{|}^{2}m_{i}}{\sum_{i}m_{i}}\right)$$

where *i* and *r*_*i*_ are the position of atom *i* with respect to the center of mass of the molecule and *m*_*i*_ is the mass of the atom. The root mean square deviation (RMSD) values were calculated using the gmx rms program (Equation (2)). The RMSD of certain atoms in a molecule with respect to a reference structure can be calculated by least-square fitting the structure to the reference structure ($t_{2}$ = 0).

(2) $$RMSD\;\left(t_{1},\;t_{2}\right)={\left[\frac{1}{M}\overset{N}{\underset{i=1}{\sum }}m_{i}{\left|\left|r_{i}\left(t_{1}\right)-r_{i}\left(t_{2}\right)\right|\right|}^{2}\right]}^{\;\frac{1}{2}}$$

where $M=\overset{N}{\underset{i=1}{\sum }}m_{i}$, and r_i_(*t*) is the position of atom *i* at time *t*.

### 2.2. Comparison between AA and CG Simulations

#### 2.2.1. Bonded Parameters

**Figure 2:**
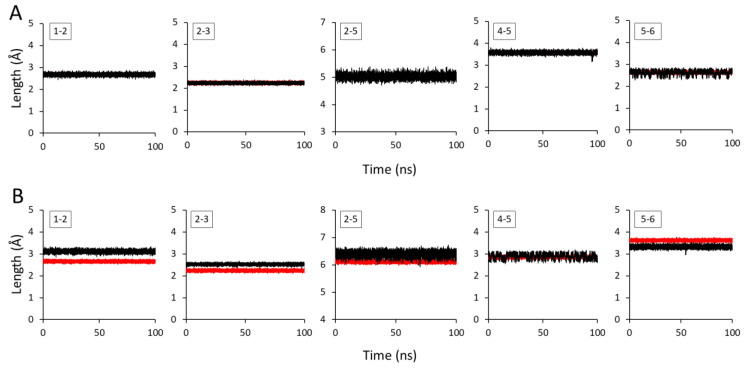
Bond lengths of all-atom (AA) and coarse-grained (CG) representations for (**A**) HA1-3 and (**B**) HA1-4. Black and red lines indicate AA and CG representations, respectively.

**Figure 3:**
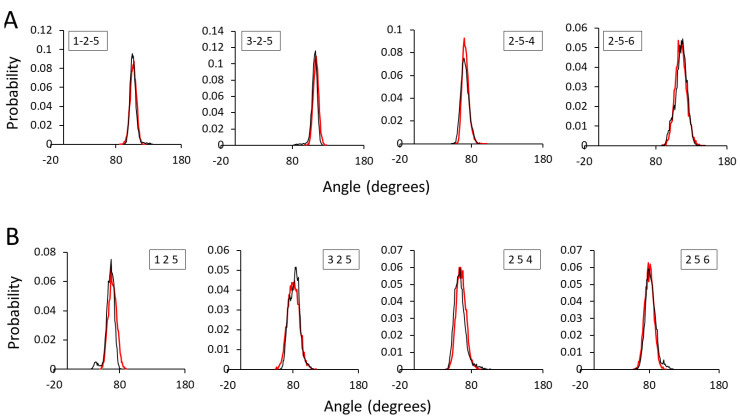
Angle distributions of AA and CG representations for (**A**) HA1-3 and (**B**) HA1-4. Black and red lines indicate AA and CG representations, respectively.

**Figure 4:**
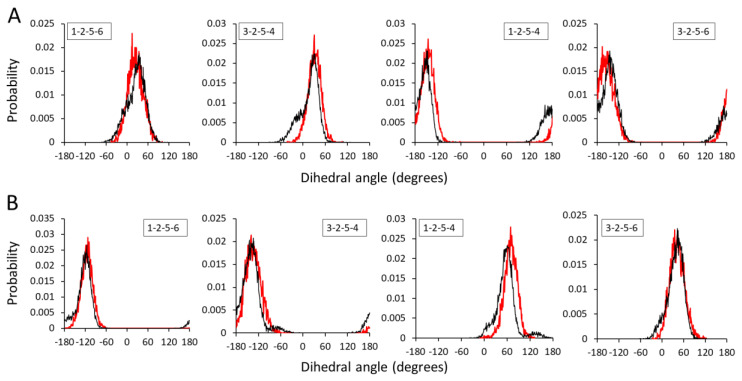
Dihedral angle distributions of AA and CG representations for (**A**) HA1-3 and (**B**) HA1-4. Black and red lines indicate AA and CG representations, respectively.

There were a total of 26 different bonded distributions for two dimers of GlcA and GlcNAc linked through HA1-3 and HA1-4 linkages (Figure 2, Figure 3, and Figure 4). The values of equilibrium bond lengths, bond angles, and dihedral angles with their respective force constants (K_a_, K_b_, and K_d_) were adjusted so that a good agreement between AA and CG bonded distributions was achieved. The details of the bonded parameters for all bond lengths, bond angles, and dihedral angles present in the HA structures obtained after many trial-and-error iterations are given in Figure 2, Figure 3, and Figure 4. The bond lengths obtained from AA and CG simulations are computed and compared in Figure 2. In the case of HA1-3, the time-averaged CG bond lengths overlapped well and showed similar lengths as AA bonds. The average bond lengths between 1-2, 2-3, 2-5, 4-5, and 5-6 were 2.67 Å, 2.23 Å, 5.04 Å, 3.56 Å, and 2.62 Å, respectively. The longest bond was observed between beads 2-5, which represents the glycosidic linkage between two monosaccharide subunits. The average bond lengths in the HA1-4 dimer showed very minute differences in AA and CG distributions (Figure 2B). The maximum difference observed between two distributions was 0.4 Å between beads 1 and 2. However, the CG distributions still matched the averaged AA distributions reasonably well, and a similar degree of discrepancy has been accepted and reported elsewhere [23]. Interestingly, there was a noticeably large difference in average bond lengths of glycosidic bonds (2-5) in HA1-3 (5.04 Å) and HA1-4 (6.21 Å) dimers, showing the importance of CG mapping two HA dimers separately.

Several adjustments of the equilibrium angle (θ_eq_ in Equation (8)) and force constant (K_a_) were made which resulted in a perfect match between AA and CG angle distributions of both HA1-3 and HA1-4 dimers (Figure 3). A slight increase in the K_a_ values generally led to a sharpening of the probability distribution peaks, as observed in angle 2-5-4 in HA1-3. A relatively large K_a_ (600) was used for angle 3-2-5 of the HA1-3 dimer, which is the angle between a P4 bead and two of main chain (MC) P2 beads. An acceptable discrepancy was observed in angle 3-2-5 of the HA1-4 dimer where the AA bond angle was 85° and the CG angle distribution was observed is at around 80°.

Comparatively larger fluctuations were observed between AA and CG dihedral angle distributions for the HA1-3 and HA1-4 dimers (Figure 4). Practically, the dihedral distributions are relatively more difficult to match among other bonded distributions, and large discrepancies have previously been reported [43]. As four beads are involved in dihedral bond formation, the most probable reason behind large fluctuations is steric hindrance among CG beads. The dihedral angles showed good agreement between AA and CG distributions in both HA1-3 and HA1-4 dimers. Several CG distributions have properly captured the location of the peaks of the distributions while only a few underestimated or overestimated certain peak heights and angles. Nonetheless, the CG distributions still match the averaged AA distributions reasonably well, and a similar degree of discrepancy has previously been reported and accepted [43,44].

#### 2.2.2. Global Properties

**Figure 5:**
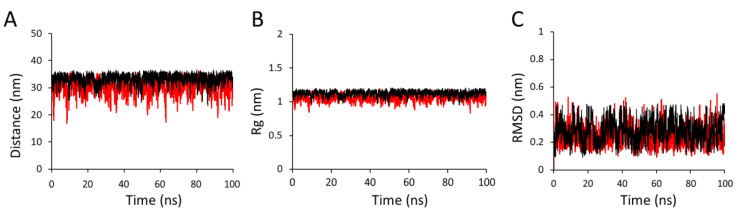
Global properties of an HA octasaccharide molecule: (**A**) End-to-end distance (R_e_), (**B**) Radius of gyration (R_g_), and (**C**) root mean square deviation (RMSD). Black and red lines indicate AA and CG representations, respectively.

**Figure 6:**
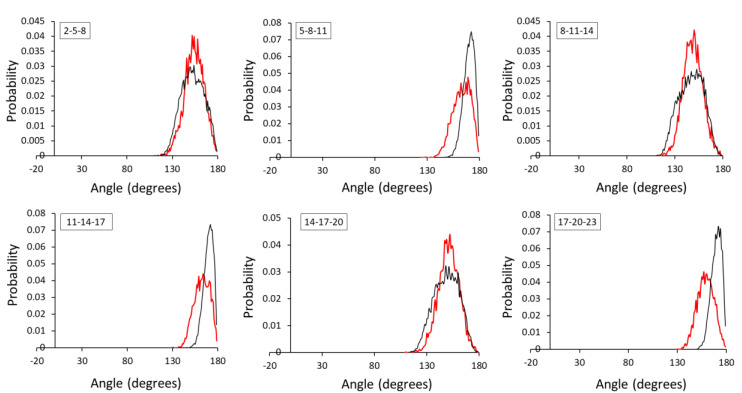
Main chain angle distributions of AA and CG representations for an HA octasaccharide molecule. Black and red lines indicate AA and CG representations, respectively.

**Figure 7:**
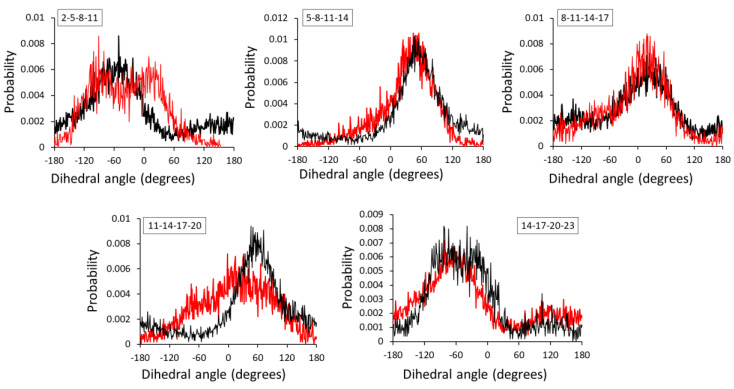
Main chain dihedral angle distributions of AA and CG representations for an HA octasaccharide molecule. Black and red lines indicate AA and CG representations, respectively.

Two important global properties of the HA polymer, namely the end-to-end distance (R_e_) and radius of gyration (R_g_), were calculated as a part of the validation and application of our CG model for HA (Figure 5). The parameters obtained from the CG modeling of HA1-3 and HA1-4 dimers were applied to model a larger HA polymer having eight monosaccharide subunits [42]. The results were compared to test if the CG model can reproduce the global properties by taking AA data as a reference. Additional MC angles and dihedral angles were applied in the case of HA octasaccharide. The distributions are indicated in Figure 6 and Figure 7. The R_e_ value of the HA polymer was calculated as the average distance between the MC beads 2 and 23 and matched with the R_e_ obtained from the analogous averaged AA simulations (Figure 2B). The R_e_ of HA polymers were well matched where the average lengths observed were 33.19 Å and 30.52 Å for AA and CG models, respectively (Figure 5A). The R_g_ of HA polymers overlapped well, with average values of 1.12 nm and 1.07 nm for AA and CG models, respectively (Figure 5B). An HA molecule eight monomers in length was used to obtain the R_g_ value, which is too short when directly compared with experimental R_g_. Therefore, we performed another simulation for HA comprised of 100 monomers in 150 mM of NaCl to correlate R_g_ values with experimental results reported by Mendichi et al. [45]. The experimental results of R_g_ followed power law as a function of molar weight in the above reference report. This experimental results estimate R_g_ of HA as 9.4 nm while we obtained a value of 8.1 nm. We believe that our simulation results are comparable to the experiment. Furthermore, the CG model of HA showed a very low range of the RMSD values of 0.005 nm to 0.498 nm, indicating an overall stiff structure of the HA (Figure 5C) [46]. The time-averaged RMSD values show a very close match, with values of 0.28 for AA and 0.31 and CG. These results confirm that the structure of CG-modeled HA is stiff in nature, which is comparable to AA HA polymer. Evidently, CG simulations of HA models have produced results that match well with the AA simulations.

### 2.3. Condensation of the Gel

#### 2.3.1. HA Gel Formation

**Figure 8:**
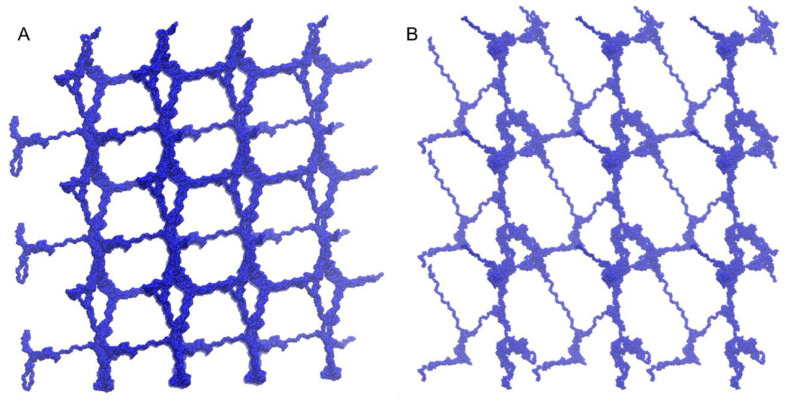
The simulation configuration of HA gel in 100 mM NaCl and: (**A**) 0 mM CaCl_2_; (**B**) 200 mM CaCl_2_.

We applied the coarse-grained model to the simulation of mesoscopic gel formation. Because HA is a negatively charged polymer, it will not aggregate unless the strong electrostatic repulsion is regulated. This is not feasible in AA because the mesoscopic phenomenon requires the consideration of multiple chains in mesoscale, and water molecules should be considered explicitly since the part of the attraction comes from the hydrophobic interaction and hydrogen bond interaction. In the simulation, we considered 16 HA chains and each was comprised of 100 monomers or 50 dimeric repeating units. The volume fraction of HA was set to be 1/1000 which corresponds to the swelling states for weakly charged polyelectrolytes [9]. In addition to the HAs and water, we added 100 mM NaCl as a physiological condition. As seen in Figure 8, we can observe that HAs are inter-connected to form a gel phase overcoming the long-range electrostatic repulsion. The crosslink is due to the short-ranged attraction from the combination of van der Waals, hydrophobic interactions, and hydrogen bonds. The result is consistent with the experimental finding of HA gel formation in physiological conditions [10], which verifies the validity of our model and its usefulness.

#### 2.3.2. Swelling of the HA Gel in the Presence of Monovalent Salt and Divalent Salt

**Figure 9:**
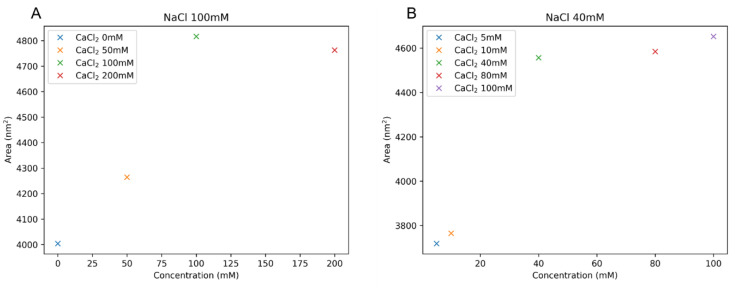
Area of HA gel when the concentrations of NaCl are (**A**) 100 mM and (**B**) 40 mM. The area is plotted as a function of CaCl_2_ concentration.

With the concentration of NaCl fixed at 100 mM, we changed the concentration of CaCl_2_ from 0 to 100 mM. Throughout the whole range of the concentration of CaCl_2_, the gel was still swollen, unlike other biopolyelectrolytes such as DNA. This is also consistent with the experimental results [10]. It is clear that NaCl screens the electrostatic interaction between HA chains. In order to investigate the change of the microscopic structure, we measured the surface area of the polyelectrolyte network. The surface area will be decreased significantly if polymers are collapsed. As seen in Figure 9A, the area increased at high CaCl_2_ concentration, but the change was not drastic. There seemed to be a slight change in the local aggregation at cross-links, but a substantial change in the network structure was not identified. 

In the experiment, the osmotic pressure was reduced with the addition of divalent salts. The polymeric contribution to the osmotic pressure of the gel corresponds to the energy stored in the volume of the network mesh (Equation (3)). That is,

(3) $$P_{osm}=\frac{k_{B}T}{l^{3}},$$

where *l* is the length of the network mesh. The length of the network mesh of the polyelectrolyte gel is proportional to $l\propto I^{1/4}$, where $I=\frac{1}{2}\sum z_{i}c_{si}^{2}$ at the condition that $c_{s+},c_{s-}\gg c$ for salt concentration $c_{s+},\;c_{s-}$ of positive and negative salt ions, and the polyelectrolyte concentration c. Then, the osmotic pressure is given by the following equation (Equation (4))

(4) $$P_{osm}\propto I^{-\frac{3}{4}}.$$

Thus, the addition of CaCl_2_ increases I which results in the decrease of the osmotic pressure in the experiments [10]. In our simulation, we did not measure the osmotic pressure directly, but we saw the consistent trend that the number of crosslinks seemed to decrease at a high salt concentration of CaCl_2_ (Figure 9).

Next we reduced the concentration of NaCl to 40 mM (Figure 9B). At this salt concentration some polyelectrolytes such as sodium polyacrylate undergo a swollen to collapsed transition under high CaCl_2_ concentration due to the ion-bridging of divalent Ca^2+^ [9]. However, our results consistently show that HA still remained in the swollen phase even for this low NaCl concentration, without establishing any ionic bridging or the resultant collapse of the gel. This stability of the HA gel under various salt concentrations is a merit for biological and medical applications. 

## 3. Conclusions

We developed a CG model of HA using the Martini scheme and compared its performance with all-atom simulations. We chose to model the HA1-3 and HA1-4 bonding disaccharide units separately. The molecular masses of the CG beads were adapted according to their AA analogs to achieve realistic simulations results. Our HA CG model was able to accurately reproduce the probability distributions for bonded interactions when compared to all-atom simulations. The obtained parameters were applied to an octasaccharide HA polymer in order to assess the performance of our CG model. The results of polymer global properties such as R_e_ and R_g_ were in good agreement with atomistic data. The structure of the HA polymer is unusually stiff in aqueous solution, which is indicated by low RMSD values during atomistic simulations. Similar results were obtained for our CG model of HA, which indicates its usefulness for modeling long HA polymers. Consequently, the CG modeling technique presented here makes long HA polymers with high molecular weights within reach in the molecular dynamics simulations approach. Here we applied our model to HA gel formation, which is usually intractable in atomistic molecular dynamics. We investigated the HA gel formation in various mixtures of NaCl monovalent salt and CaCl_2_ divalent salt, from physiological to lab conditions. We found that HA formed a stable swollen gel over the salt concentrations we investigated, which is consistent with the experimental results. This is a distinguishable feature of HA from regular proteins, whose backbones are usually hydrophobic. This property is advantageous for bioengineering applications of HA. We believe these developed procedures could serve as a useful reference for further coarse-grained modeling of other polyelectrolytes in order to enable mesoscale simulations with large molecules. Our model developed here can be used in conjunction with other Martini models, and can be further expanded to model various HA polymers and their modified polyelectrolytes, for example, the process of physical crosslinking of HA in the presence of phospholipids (HA–PL complex) in the lubrication of articular cartilage [47,48], self-assembly of polymeric nanoparticles in drug delivery [49,50], novel HA-based biocompatible and biodegradable hydrogels for in vitro cell culture [2,51], cartilage tissue engineering [3], and the charge-based purification of protein drugs [4].

## 4. Materials and Methods

### 4.1. Model

A typical Martini model applies a mapping of four heavy atoms and associated hydrogens to one (4:1 mapping) CG bead [52]. Each CG bead is assigned a bead type based on thermodynamic properties obtained from experiments and simulations of its AA molecular analog. Different bead sizes can be used including a “standard” bead representing 4:1 mapping. “Small” and “tiny” beads with 3:1 or 2:1 mapping are typically used to model ring-like structures and base stacking in nucleic acids. The mapping strategy is flexible and can be adjusted depending on one’s need such that higher or lower or even fractional mapping ratios can be used for different biomolecules [53]. Furthermore, Martini models are kept simple by assigning only four main types of interaction sites or bead types such as polar (P), nonpolar (N), apolar (C), and charged (Q) beads. The Martini “standard” CG beads types can be further subdivided to capture their different chemical natures. Depending on their polarity, polar and apolar bead types are subdivided into five levels from 1 to 5 (polarity low to high). Whereas, nonpolar and charged beads are subdivided based on their hydrogen bonding (d (donor), a (acceptor), da (both), 0 (none)) capability. The parameters for nonbonded interactions are determined by the assigned bead type and size. The mass of CG beads can be adapted according to its AA analog in order to achieve realistic simulations [52]. For bonded parameters, two sets of simulations (AA and CG) are run and probability distributions of bond lengths, bond angles, and dihedral angles are compared and tuned by trial and error until a good match is obtained. 

### 4.2. CG Mapping

The HA molecule was mapped based on the Martini model for carbohydrates where disaccharides are modeled as two three-bead units connected by a single bond mimicking the glycosidic linkage in carbohydrates [43]. Such additional bonds are used to connect multiple monosaccharides units to construct oligosaccharide and polysaccharide CG mappings. We parameterized HA1-3 and HA1-4 subunits separately (Figure 1A,B). Two types of Martini CG bead types were assigned: polar (P type) and charged (Q type) (Table 1 and Table 2). In the case of GlcA, the charged bead type was Qa, while the remaining two were assigned P2 and P4 bead types. In GlcNAc, all three beads were polar and included two P2 and one P1 bead type. This type of mapping was carried forward to a larger molecule of HA with eight monosaccharide subunits whose structure (PDB ID: 2BVK) is available at the RCSB database [42]. We denoted the central beads of both monosaccharide units 2, 5, 8, 11, 14, 17, 20, and 23 as main chain (MC), atoms which are represented by the P2 bead type (Figure 1C).

### 4.3. Non-Bonded Interactions

The non-bonded interactions were mainly represented by van der Waals interactions and electrostatic interactions (Equation (5)). The van der Waals interactions were described by the 12–6 Lennard-Jones (LJ) potential energy function given as

(5) $$V_{LJ}=4\varepsilon_{ij}\left[{\left(\frac{\sigma_{ij}}{r_{ij}}\right)}^{12}-{\left(\frac{\sigma_{ij}}{r_{ij}}\right)}^{6}\right]$$

where *σ_ij_* represents the closest distance of approach between two particles while the strength of their interaction is given by *ε_ij_*, and the LJ parameters *σ_ij_* and *ε_ij_* were determined by the assigned bead type. In case of the charged beads, the electrostatic interaction was represented by the Columbic potential energy function (Equation (6)) given as

(6) $$V_{el}=\frac{q_{i}q_{j}}{4\pi \varepsilon_{0}\varepsilon_{r}r_{ij}}$$

where *q_i_* and *q_j_* denote the charges of CG beads *i* and *j*, respectively, *ε_r_* is the relative dielectric constant of the solvent, and *ε*_0_ is the permittivity of free space.

### 4.4. Bonded Interactions

The bonded interactions mainly constituted bond lengths, bond angles, and dihedral angles. The harmonic potential *V_b_* is used to model bond lengths (Equation (7)). Bond length parameter *r_ij_* denotes the distance between two bonded beads *i* and *j*, *r_eq_* is the equilibrium bond length, and K_b_ represents the force constant.

(7) $$V_{b}=\frac{1}{2}K_{b}{\left(r_{ij}-r_{eq}\right)}^{2}$$

A cosine type harmonic potential denoted by *V_a_* was used to model bond angles (Equation (8)), where *θ_ijk_* is the angle between three consecutive beads *i*, *j*, and *k*, *θ_eq_* is the equilibrium bond angle, and K_a_ is the force constant.

(8) $$V_{a}=\frac{1}{2}K_{a}\left(cos\left(\theta_{ijk}\right)-\cos(\theta_{eq}\right){)}^{2}$$

*V_d_* represents a sum of periodic potentials where *φ_ijkl_* is the dihedral angle between sequential beads *i*, *j*, *k*, and *l* (Equation (9)). The force constant for dihedral angles is given by *K*_d_, number of peaks in this potential by *n*, and *φ_d_* specifies the location of peaks in periodic potential.

(9) $$V_{d}=K_{d}\left(1+cos\left(n\varphi_{ijkl}-\varphi_{d}\right)\right)$$

### 4.5. AA Simulations 

AA simulations were run on three different molecules: two HA dimers HA1-3 and HA1-4, and an HA octasaccharide, independently. The simulations were carried out with CHARMM36 force field using Groningen machine for chemical simulations (GROMACS 2018) software [54]. The topology files for all ligands were generated using the CHARMM General Force Field (CGenFF) program implemented in CHARMM-GUI webserver [55]. Each system contained one HA in dodecahedron water box of thickness 10 Å containing TIP3P waters with an appropriate number of counter-ions added to neutralize the system. The initial structures were relaxed through 50,000 steps of energy minimization with a maximum force of 1000 kJ/mol using steepest descent algorithm. Thereafter, a two-step equilibration procedure including a constrained NVT simulation at 300 K and a constrained NPT simulation at 300 K and 1 bar was performed. The temperature was maintained using a V-rescale thermostat with a time constant of 0.1 ps. The Parrinello–Rahman barostat was used to maintain the pressure with a time constant of 2 ps and compressibility of 4.5 × 10^−5^ bar^−1^. The bonds were constrained using the LINCS algorithm. An unrestrained production run was carried out for 100 ns under NPT conditions using periodic boundary condition applied in all directions. The equations of motion were integrated using the leapfrog algorithm with a time step of 2 fs. The long-range electrostatic interactions were calculated using the particle mesh Ewald (PME) method. Nonbonded short-range interactions were cut off at 1.0 nm.

### 4.6. CG Simulations

The average structures were taken from the last 50 ns of AA simulations. These equilibrated configurations were used to generate initial structures of HAs for CG simulations using the mapping scheme specified in the CG mapping section described above. The same temperature and pressure conditions were maintained similar as in the AA simulations. Solvation was done with a standard non-polarizable water model and counterions were added to neutralize the system. Further, the energy minimization was followed by a constrained NPT equilibration of 1 ns, excluding NVT. The NVT simulation was not necessary prior to the NPT simulation as the low-amplitude high-frequency fluctuations were removed from the potential energy surfaces (PES) in the CG models [14]. The cut-off value of 1.1 nm was used for the LJ potential and short-range nonbonded interactions. The dielectric constant of 15 was used for water. A comparatively larger time step of 10 fs was used in all CG simulations compared to AA simulations. A production run of 100 ns was completed under the unconstrained NPT simulation with periodic boundary conditions applied in all directions.

## Figures and Tables

**Table 1:** Assignment of Martini CG bead types for the HA1-3 disaccharide.

Bonding	Structure	No.	Bead Name	Bead Type
GlcA1-3GlcNAc	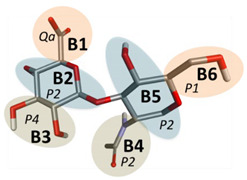	1	B1	Qa
		2	B2	P2
		3	B3	P4
		4	B4	P2
		5	B5	P2
		6	B6	P1

**Table 2:** Assignment of Martini CG bead types for HA1-4 disaccharide.

Bonding	Structure	No.	Bead Name	Bead Type
GlcNAc1-4GlcA	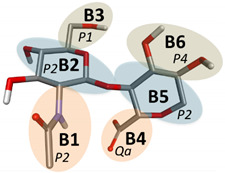	1	B1	P1
		2	B2	P2
		3	B3	P2
		4	B4	Qa
		5	B5	P2
		6	B6	P4
